# Oral Janus Kinase Inhibitor for the Treatment of Rheumatoid Arthritis: Tofacitinib

**DOI:** 10.1155/2013/357904

**Published:** 2013-07-21

**Authors:** Han Ni, Soe Moe, Kay Thi Myint, Aung Htet

**Affiliations:** ^1^Internal Medicine, SEGi University, No. 9 Jalan Teknologi, Taman Sains Selangor, Kota Damansara, PJU 5, 47810 Petaling Jaya, Selangor, Malaysia; ^2^Community Medicine, Melaka Manipal Medical College, Jalan Batu Hampar, Bukit Baru, Malacca 75150, Malaysia; ^3^Ophthalmology, Melaka Manipal Medical College, Jalan Batu Hampar, Bukit Baru, Malacca 75150, Malaysia; ^4^Diagnostic Radiology, Defense Service General Hospital, Naypyitaw, Myanmar

## Abstract

Since the introduction of immune modulators in the treatment of rheumatoid arthritis (RA), there has been hope that orally effective biologic agents would be developed. Tofacitinib, a Janus kinase inhibitor, has become the first oral biologic to receive approval for use in active RA patients. This paper reviews the efficacy and safety profile of Tofacitinib at dosages of 5 mg and 10 mg twice daily. Remarkable improvement in terms of ACR 20 response and HAQ-DI score was noted at month 3 and month 6. DAS 28-4 ESR < 2.6 achievement was noticeably obvious at month 6 for both dosages. No significant serious adverse events, serious infections, neutropenia, or anaemia were observed compared to placebo. In fact, Tofacitinib 5 mg was even found to have significant protective effect of anaemia in the meta-analysis (*P* = 0.004). Tofacitinib has a noticeable efficacy in controlling disease activity in RA with a manageable safety profile. However, longer studies are needed for its long-term safety profile.

## 1. Introduction

Rheumatoid arthritis (RA) is a common immune-mediated systemic disorder, characterized by inflammatory polyarthritis affecting synovium of joints, tendons, and extra-articular sites. It is progressive and leads to joint erosions and deformities, causing premature mortality, functional impairment, and reduced quality of life [1].

The prevalence of RA remains constant at 0.5–1.0% among various population group [2, 3]. The prevalence is generally lower in developing countries [4]. In 2005, 1.5 million adults of more than 18 years (0.6%) in the United States were estimated to have RA [5]. HLA DRB1 allele is the major genetic risk factor of RA around the world [2].

Conventionally, RA was treated with “Pyramid” approach, where disease modifying antirheumatic drug (DMARD) was deferred until advanced stage. In 1960s and 1970s, gold and penicillamine were the only DMARDs used for RA [6]. However, in 1980s, methotrexate was found to retard or even prevent bone erosions [7]. This has led to dramatic changes in the treatment of RA, with early aggressive use of DMARDs within the first few months of diagnosis, and methotrexate becomes the first line DMARD in RA [6].

In the pathogenesis of rheumatoid arthritis, various inflammatory mediators are found to be involved, among which tumour necrosis factor (TNF) *α* is the main agent. New drugs targeting these inflammatory mediators have changed the prognosis and outcome of this chronic debilitating disease. Early initiation of DMARDs, either nonbiologic or biologic, has decreased the morbidity of this condition [8, 9].

Thus, the recent 2012 American College of Rheumatology (ACR) guidelines on management of RA recommends the use of DMARDs in early RA of less than six months duration as monotherapy for patients with low disease activity and combination therapy for moderate or high disease activity. It also recommends the use of anti-TNF biologics with or without methotrexate for early RA with high disease activity and poor prognostic factors [10].

## 2. Role of Biologic Agents in Rheumatoid Arthritis

Interaction between adaptive and innate immune systems is central in the synovial inflammation. In rheumatoid arthritis, the synovium has abundant myeloid cells and plasmacytoid dendritic cells that express cytokines such as interleukin-12, 15, 18, and 23 and HLA class II molecules as well as costimulatory molecules that play a role in antigen presentation and T-cell activation [11]. Macrophages are central effectors of synovitis by releasing a variety of inflammatory cytokines, with TNF *α* and IL-6 being the most important mediators, ultimately leading to breakdown of extracellular matrix of cartilage and bone [11, 12]. B cells also play a role in autoantigen presentation and cytokine production (e.g., interleukin-6, TNF-*α*, and lymphotoxin-*β*) [11].

Approved biologic agents used in RA include cytokine inhibitors of TNF alpha (adalimumab, etanercept, infliximab, certolizumab pegol, and golimumab), IL-6 receptor (tocilizumab), IL-1 (anakinra), cell depleting agent targeting CD 20 of B cells (rituximab), and costimulation blocker of cytotoxic T lymphocyte antigen-4 (abatacept) [11, 13, 14].

However, the limitation of these biologics which requires parenteral administration, either intravenously or subcutaneously, has necessitated the development of new orally effective small molecules for the treatment of rheumatoid arthritis.

## 3. JAK Inhibitors/Tofacitinib

Janus kinase-signal transducer and activator of transcription (JAK-STAT) pathway was first discovered twenty years ago to play an essential role in interferon-dependent cytoplasmic signaling of inflammatory response [7, 25, 26]. JAK family consists of cytosolic tyrosine kinases that regulate cytokine-mediated leucocyte maturation and activation, cytokine production, and immunoglobulin production [11, 27, 28]. There are four JAKs: JAK1, JAK2, JAK3, and tyrosine kinase 2 (TYK2) which are selectively associated with the cytoplasmic domains of various cytokine receptors such as IL-2, IL-6, IL-7, IL-12, interferon erythropoietin, and growth hormone [7, 27, 29]. After the ligation of cytokines or growth factors with the respective receptors, JAKs become activated and convey signals to the cytosolic STATs that migrate into the nucleus to promote gene expression [7, 26–29].

JAK3 and TYK2 are primarily involved in immune responses, while JAK1 and JAK2 have various functions ranging from host defense and hematopoiesis to growth and neural development [26, 29].

Tofacitinib (CP-690,550) is the most studied JAK inhibitor in RA, which mainly inhibits JAK1 and JAK3 with lesser extent on JAK2. Since JAK2 plays a role in haematopoiesis, this selectivity is advantageous for Tofacitinib with lesser haematological adverse effects [7, 29]. In animal models of experimental arthritis, Tofacitinib was shown to be effective in preventing cartilage damage [7]. Subsequent studies done in human RA patients also reveal promising results with improvement in disease activity. In this review, we will analyze the efficacy and safety of Tofacitinib in active rheumatoid arthritis patients.

## 4. Material and Methodology

We identified published randomized controlled trials on Tofacitinib in RA patients by searching the PubMed database (until May 2013) using the search terms “Tofacitinib” OR “Janus Kinase inhibitor” AND “Rheumatoid arthritis” AND “randomiz/sed controlled trial.” We also searched the reference lists of all included studies, US Food and Drug Administration (FDA) [30], pharmaceutical websites (Pfizer), and proceedings and abstracts of rheumatology conferences. We included studies of adult RA patients (18 years or older) diagnosed by the ACR 1987 revised criteria (Table 4). We only included the active disease defined as the presence of 6 or more tender or painful joints (out of 68 specific joints examined) and 6 or more swollen joints (out of 66 specific joints examined) and had either Westergren erythrocyte sedimentation rate (ESR) of more than 28 mm per hour or C-reactive protein (CRP) level of more than 7 mg per liter. These patients are either methotrexate/DMARD naive or with incomplete response to prior biologic or nonbiologic DMARDs who continue taking methotrexate, NSAIDs, or steroids at the stable dosage. We excluded studies with other kinase inhibitors or oral biologic agents. We analyzed the ACR 20 response at month 3 or month 6; Disease Activity score for 28-joint counts based on the erythrocyte sedimentation rate (DAS 28-4 ESR) < 2.6 at month 3 or month 6; change in Health Assessment Questionnaire-Disability Index (HAQ-DI) score from baseline at month 3 for the efficacy of Tofacitinib; serious adverse events, serious infections, and cytopenia for safety. ACR 20 response is defined as at least 20% improvement in both swollen and tender joint counts and three out of the following five variables: patient and evaluator global disease activity, pain assessment, functional disability, and acute-phase reactants (sedimentation rate or C-reactive protein).

## 5. Data Extraction and Management

The review authors assessed the search results according to the eligibility criteria for inclusion in this review and extracted data from the included trials. The data were entered into the Review Manager Software version 5.2 for statistical analysis. Mantel-Haenszel odds ratio (OR) with 95% confidence interval (CI) is used for meta-analysis of dichotomous outcome data.

## 6. Results

### 6.1. Description of Studies

Our search strategy yielded 14 potentially relevant results, among which nine (four phase 3 trials [15, 16, 18, 19] and five phase 2 trials [20–24]) fulfill our selection criteria. Further search from the conference proceedings indentified the abstract of the fifth phase 3 trial [17]. A total of ten RCTs met our inclusion criteria (Table 1), but two of them were not involved in the data analysis because their outcome measures did not meet our criteria. Both of them were only six-week studies [23, 24].

### 6.2. Efficacy of Tofacitinib

**Figure 1:**
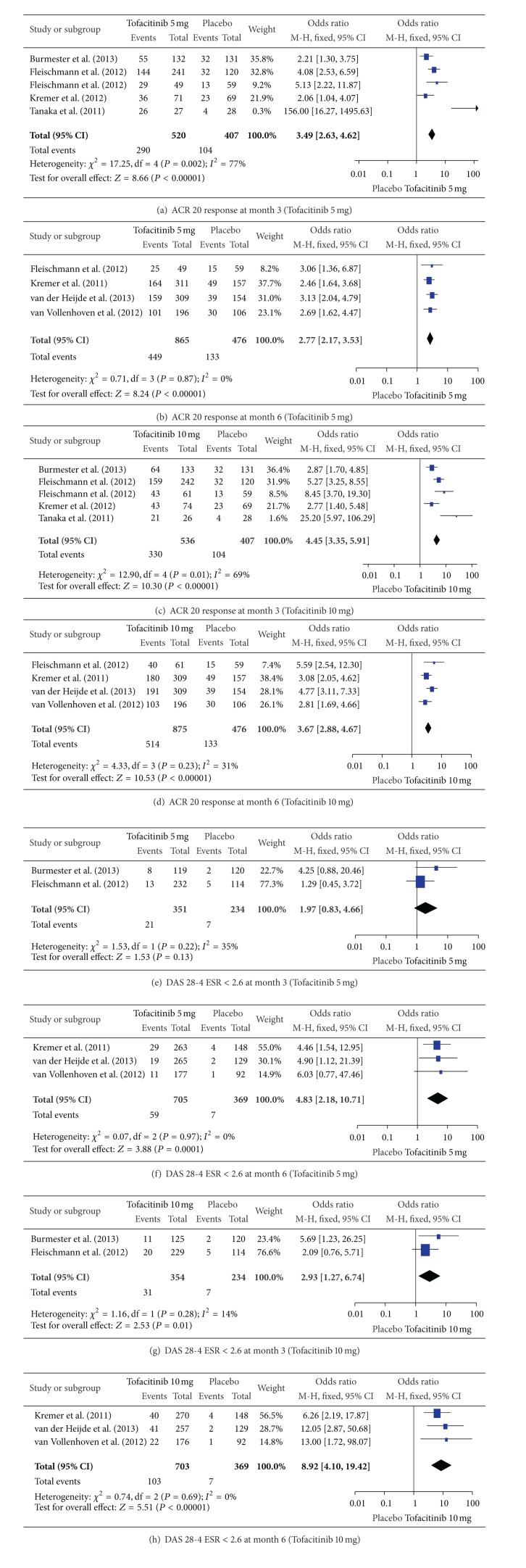
Forest plots showing ACR 20 response and DAS 28-4 ESR < 2.6 at month 3 and 6 with Tofacitinib 5 mg and 10 mg twice daily.

Meta-analysis of the efficacy outcome measures showed that the ACR 20 response at month 3 and month 6 was significantly better with Tofacitinib at both 5 mg and 10 mg twice daily compared to placebo (Figures 1(a)–1(d)). The proportion of active RA patients whose DAS 28-4 ESR < 2.6 at month 6 was significantly higher for Tofacitinib at both 5 mg and 10 mg. However, at month 3, DAS 28-4 ESR < 2.6 achievement was not significant with 5 mg dosage while it was significant for 10 mg twice daily dosage compared with placebo (Figures 1(e)–1(h)).

Consistent and statistically significant improvement in HAQ-DI score from the baseline at month 3 with Tofacitinib 5 mg and 10 mg compared to placebo was noted across all the included studies (Table 2).

### 6.3. Safety of Tofacitinib

No statistically significant difference was noted for safety outcome measures with Tofacitinib 5 mg or 10 mg at month 3 and 6 compared to placebo (Table 3). There were no reported cases of life-threatening neutropenia in all the included phase 3 studies [15–19]. Occurrence of both mild neutropenia and moderate to severe neutropenia were not statistically higher among the Tofacitinib group.

Interestingly, Tofacitinib 5 mg twice daily was found to have a significant protective effect compared to placebo (*P* = 0.004) regarding anaemia (decrease in haemoglobin of −1 to −3 g/dL) at month 0–3 (Table 3).

Elevation of LDL cholesterol was reported to be significant in both Tofacitinib 5 mg and Tofacitinib 10 mg groups compared to placebo in two phase 3 trials {(*P* < 0.001) [16] and (*P* < 0.0001) [15]}.

## 7. Discussion

Tofacitinib is the first orally active biologic agent approved by US FDA in November 2012 for use in moderate to severe active adult RA patients with prior inadequate response to, or who are intolerant of, methotrexate either as monotherapy or combined with methotrexate. Combination therapy with other biologics is not recommended [31]. It is a potent inhibitor of JAK 1 and JAK 3 which binds to *β* and *γ* chains of cytokine receptor, respectively, to which inflammatory mediators such as IL-2, IL-4, IL-7, IL-9, IL-15, and IL-21 attach to initiate synovial inflammation in RA [32, 33].

In this review, we assessed the efficacy and safety of Tofacitinib in active RA patients by analyzing the results of randomized placebo-controlled clinical trials. Ten studies are identified, eight of which are included in data analysis with a total of 4347 participants. The trials reviewed were of a similar design with at least three arms: placebo, Tofacitinib 5 mg twice daily, and Tofacitinib 10 mg twice daily with duration of 3 to 24 months. However, the results are only available till month 12. Longer clinical trials with results are required for full evaluation of long-term efficacy and safety of Tofacitinib.

There are three primary efficacy outcome measures of this review: ACR 20 response, DAS 28-4 ESR < 2.6, and improvement in HAQ-DI score. Two doses of Tofacitinib 5 mg and 10 mg twice daily produced statistically significant ACR 20 response at month 3 and month 6 compared to placebo (*P* < 0.00001). For DAS 28-4 ESR < 2.6, Tofacitinib 5 mg did not result in significant change at month 3 (*P* = 0.13). However, at month 6, 5 mg twice daily dosage provided significant improvement (*P* < 0.0001). Higher dosage of 10 mg twice daily showed significance since the early period of month 3 (*P* = 0.01). More significant improvement in DAS 28-4 ESR < 2.6 achievement was noted at month 6 (*P* < 0.00001). Improvement in HAQ-DI score from the baseline was also consistently better for oral Tofacitinib 5 mg and 10 mg twice daily compared with placebo. These promising efficacy results combined with its oral route of administration would make Tofacitinib a better choice as a biologic for active RA patients not responding to methotrexate or other DMARDs.

Nevertheless, trials on head to head comparison between Tofacitinib and other approved biologic agents are lacking. Only one trial in this review included subcutaneous adalimumab 40 mg every 2 weeks as one arm in the study design [19]. Further randomized controlled trials comparing the efficacy of Tofacitinib with other approved biologic agents for RA are recommended.

Like other biologic agents, Tofacitinib has the risk of infections, cancer, lymphomas, and cytopenias. Active tuberculosis (TB) is one of the serious infections that usually occur with biologic agents, and US FDA recommends to rule out latent TB prior to initiation of therapy and to monitor for active TB during treatment period [34]. In this meta-analysis, the risk of serious adverse events and serious infections with Tofacitinib 5 mg and 10 mg twice daily was not significantly higher in relation to the placebo group at month 3. Similarly, these safety outcome measures are not statistically different among 5 mg and 10 mg dosages at month 6 of therapy. Common adverse events reported in individual phase 3 studies were infections, headache, nausea, vomiting, and diarrhea. Infections included bronchitis, nasopharyngitis, upper respiratory tract infection, pneumonia, cellulitis, urinary tract infection, and herpes zoster [35].

Cytopenias, especially decrease in neutrophil count and haemoglobin, were reported among Tofacitinib-treated RA patients. Occurrence of mild neutropenia (1500–1999 cells/mm^3^) was higher than moderate to severe neutropenia (500–1499 cells/mm^3^) with Tofacitinib group, which was not statistically significant in comparison with placebo group on meta-analysis of the studies. There were no reported cases of life-threatening neutropenia across the studies. Reduction in haemoglobin by 1–3 g/dL from the baseline is expected to be higher among Tofacitinib group compared to placebo. However, meta-analysis of the data across the included studies revealed an interesting result of protective effect on anaemia by Tofacitinib, compared to placebo group with 5 mg dosage (*P* = 0.004). Detailed observation of the placebo group was needed before we conclude the protective effect of Tofacitinib 5 mg in anaemia. Nonetheless, it can be confidently concluded that Tofacitinib does not increase the risk of anaemia with both 5 mg and 10 mg doses. Lesser inhibition of JAK 2, which is responsible for erythropoiesis, by Tofacitinib might explain this finding.

Other reported side effects of Tofacitinib include hypercholesterolaemia and rise in liver enzymes (which rarely exceeds beyond 3 times upper limit) and serum creatinine. In a meta-analysis of safety profile of protein kinase inhibitors, hypercholesterolaemia was reported to be significantly higher with Tofacitinib than the comparator group with dose-related increase in mean serum total cholesterol, HDL, and LDL at week 6 [36]. Nevertheless, larger trials with longer duration of study and postmarketing adverse event reporting are necessary for monitoring of long-term safety of this new effective antirheumatic drug.

## 8. Conclusion

In summary, Tofacitinib is an effective oral biologic agent with manageable safety profile for active RA patients with incomplete response to other DMARDs.

## Figures and Tables

**Table 1:** Summary of published studies on Tofacitinib.

Study	Phase	Duration	Participants	Intervention	Primary Outcome
Burmester et al. [15]	3	6 months	Moderate to severe RA Patients with inadequate response to TNF*α* inhibitors (*N* = 399)	Tofacitinib 5 mg bd; Tofacitinib 10 mg bd; placebo all with methotrexate	ACR 20 response at month 3; DAS 28-4 ESR < 2.6 at month 3; HAQ-DI at month 3 (change from baseline)

Fleischmann et al. [16]	3	6 months	Active RA patients receiving stable doses of antimalarial agents Prior inadequate response to at least one DMARD (biologic or nonbiologic) (*N* = 611)	Tofacitinib 5 mg bd; Tofacitinib 10 mg bd; placebo for 3 months followed by Tofacitinib 5 mg bd; placebo for 3 months followed by Tofacitinib 10 mg bd	ACR 20 response at month 3; DAS 28-4 ESR < 2.6 at month 3; HAQ-DI at month 3 (change from baseline)

Kremer et al. [17]	3	12 months	Active RA Patients with inadequate response to ≥1 DMARD (*N* = 792)	Tofacitinib 5 mg bd; Tofacitinib 10 mg bd; placebo	ACR 20 response at month 6; DAS 28-4 ESR < 2.6 at month 6; HAQ-DI at month 3 (change from baseline)

van der Heijde et al. [18]	3	24 months	Active RA patients receiving background methotrexate (*N* = 797)	Tofacitinib 5 mg bd; Tofacitinib 10 mg bd; placebo followed by Tofacitinib 5 mg bd; placebo followed by Tofacitinib 10 mg bd	ACR 20 response at month 6; DAS 28-4 ESR < 2.6 at month 6; HAQ-DI at month 3 (change from baseline)

van Vollenhoven et al. [19]	3	12 months	Active RA patients receiving stable doses of methotrexate (*N* = 717)	Tofacitinib 5 mg bd; Tofacitinib 10 mg bd; adalimumab 40 mg every 2 weeks; placebo	ACR 20 response at month 6; DAS 28-4 ESR < 2.6 at month 6; HAQ-DI at month 3 (change from baseline)

Fleischmann et al. [20]	2b	24 weeks	Active RA Patients with inadequate response to DMARD (*N* = 384)	Tofacitinib 1 mg, 3 mg, 5 mg, 10 mg, or 15 mg bd; adalimumab 40 mg every 2 weeks for 6 injections followed by Tofacitinib 5 mg bd; placebo	ACR 20 response at week 12

Kremer et al. [21]	2b	24 weeks	Active RA patients receiving stable doses of methotrexate with inadequate response to methotrexate alone (*N* = 507)	Tofacitinib 20 mg daily; Tofacitinib 1 mg, 3 mg, 5 mg, 10 mg, or 15 mg bd; placebo	ACR 20 response at week 12

Tanaka et al. [22]	2	12 weeks	Active RA patients receiving stable doses of methotrexate with inadequate response to methotrexate alone (*N* = 140)	Tofacitinib 1 mg, 3 mg, 5 mg, and 10 mg bd; placebo	ACR 20 response at week 12

Kremer et al. [23]	2a	6 weeks	Active RA Patients with inadequate or toxic response to methotrexate, etanercept, infliximab, or adalimumab (*N* = 264)	Tofacitinib 5 mg, 15 mg, and 30 mg bd; placebo	ACR 20 response at week 6

Coombs et al. [24]	2	6 weeks	Moderate to severe active RA Patients with inadequate response to methotrexate or a TNF*α* inhibitor (*N* = 264)	Tofacitinib 5 mg, 15 mg, and 30 mg bd; placebo	Visual analogue scale scores at week 6; HAQ-DI (change from baseline) and short form-36 (SF-36) at week 6

**Table 2:** Change in HAQ-DI score at month 3 (from baseline).

Study	Total (*N*)	Tofacitinib 5 mg bd	Tofacitinib 10 mg bd	Placebo	Adalimumab 40 mg every 2 weeks
Burmester et al. [15]	399	−0.43^§^	−0.46^§^	−0.18	—
Fleischmann et al. [16]	611	−0.50**	−0.57**	−0.19	—
Kremer et al. [17]	792	−0.46^§^	−0.56^§^	−0.21	—
van der Heijde et al. [18]	797	−0.40^#^	−0.54^§^	−0.15	—
van Vollenhoven et al. [19]	717	−0.55**	−0.61**	−0.24	−0.49**
Fleischmann et al. [20]	384	−0.51*	−0.66^§^	−0.25	—
Kremer et al. [21]	507	−0.49**	−0.39*	−0.16	—
Tanaka et al. [22]	140	−0.49**	−0.57**	−0.05	—

*Significant *P* < 0.05; **significant *P* < 0.001; ^§^significant *P* < 0.0001; ^#^significance not declared.

**Table 3:** Analysis of safety profile of Tofacitinib.

Outcome	Number of studies	Number of participants	Odds ratio [Confidence Interval]	*P*-value
Serious adverse events with Tofacitinib 5 mg versus placebo at month 0–3	5 [15–19]	1891	0.80 [0.47, 1.35]	0.39
Serious adverse events with Tofacitinib 10 mg versus placebo at month 0–3	5 [15–19]	1896	0.77 [0.45, 1.31]	0.33
Serious infections with Tofacitinib 5 mg versus placebo at month 0–3	4 [15, 16, 18, 19]	1423	1.91 [0.31, 11.70]	0.49
Serious infections with Tofacitinib 10 mg versus placebo at month 0–3	4 [15, 16, 18, 19]	1418	2.10 [0.44, 9.94]	0.35
Serious adverse events with Tofacitinib 5 mg versus 10 mg at month 3–6	5 [15–19]	2427	1.28 [0.80, 2.03]	0.30
Serious adverse events with Tofacitinib 5 mg versus 10 mg at month 6–12	2 [18, 19]	1042	1.53 [0.79, 2.97]	0.21
Serious infections with Tofacitinib 5 mg versus 10 mg at month 3–6	4 [15, 16, 18, 19]	1797	1.62 [0.67, 3.94]	0.28
Serious infections with Tofacitinib 5 mg versus 10 mg at month 6–12	2 [18, 19]	1042	0.74 [0.16, 3.31]	0.69
Mild neutropenia (1500–1999 cells/mm^3^) with Tofacitinib 5 mg versus placebo at month 0–3	4 [15, 16, 18, 19]	1308	1.55 [0.64, 3.77]	0.33
Mild neutropenia (1500–1999 cells/mm^3^) with Tofacitinib 10 mg versus placebo at month 0–3	4 [15, 16, 18, 19]	1312	1.97 [0.83, 4.67]	0.12
Moderate to severe neutropenia (500–1499 cells/mm^3^) with Tofacitinib 5 mg versus placebo at month 0–3	4 [15, 16, 18, 19]	1308	3.26 [0.71, 14.95]	0.13
Moderate to severe neutropenia (500–1499 cells/mm^3^) with Tofacitinib 10 mg versus placebo at month 0–3	4 [15, 16, 18, 19]	1312	2.99 [0.52, 17.02]	0.22
Anaemia (decreased haemoglobin −1 to −3 g/dL) with Tofacitinib 5 mg versus placebo at month 0–3	4 [15, 16, 18, 19]	1339	0.56 [0.38, 0.83]	0.004*
Anaemia (decreased haemoglobin −1 to −3 g/dL) with Tofacitinib 10 mg versus placebo at month 0–3	4 [15, 16, 18, 19]	1337	1.05 [0.74, 1.48]	0.80

*Significant.

**Table 4:** Revised ACR 1987 criteria for diagnosis of rheumatoid arthritis (RA).

Criterion	Definition
(1) Morning stiffness	Morning stiffness in and around the joints, lasting at least 1 hour before maximal improvement
(2) Arthritis of 3 or more joint areas	At least 3 joint areas simultaneously have had soft tissue swelling or fluid (not bony overgrowth alone) observed by a physician. The 14 possible areas are right or left PIP, MCP, wrist, elbow, knee, ankle, and MTP joints
(3) Arthritis of hand joints	At least 1 area swollen (as defined above) in a wrist, MCP, or PIP joint
(4) Symmetric arthritis	Simultaneous involvement of the same joint areas (as defined in 2) on both sides of the body (bilateral involvement of PIPs, MCPs, or MTPs is acceptable without absolute symmetry)
(5) Rheumatoid nodules	Subcutaneous nodules over bony prominences, extensor surfaces, or juxtaarticular regions, observed by a physician
(6) Serum rheumatoid factor	Demonstration of abnormal amounts of serum rheumatoid factor by any method for which the result has been positive in <5% of normal control subjects
(7) Radiographic changes	Radiographic changes typical of rheumatoid arthritis on posteroanterior hand and wrist radiographs, which must include erosions or unequivocal bony decalcification localized in or most marked adjacent to the involved joints (osteoarthritis changes alone do not qualify)

Note: criteria 1 through 4 must have been present for at least 6 weeks. Rheumatoid arthritis is defined by the presence of 4 or more criteria, and no further qualifications (classic, definite, or probable) or list of exclusions are required.

## Abbreviations

ACR: American college of rheumatology
CDC: Center for disease control
CRP: C-reactive protein
DAS: Disease activity score
DMARD: Disease modifying antirheumatic drug
ESR: Erythrocyte sedimentation rate
HAQ-DI: Health Assessment Questionnaire-Disability Index
HLA: Human leucocyte antigen
IL: Interleukin
JAK: Janus kinase
NSAID: Nonsteroidal anti-inflammatory drug
RA: Rheumatoid arthritis
STAT: Signal transducer and activator of transcription
TB: Tuberculosis
TNF: Tumour necrosis factor
US FDA: United States Food and Drug Administration
WHO: World Health Organization
YLD: Years lived with disability.
